# Topical Dorzolamide as Adjunctive Treatment With Intravitreal Bevacizumab in Bilateral Diabetic Macular Edema

**DOI:** 10.7759/cureus.54829

**Published:** 2024-02-24

**Authors:** Ata Sadr, Meisam Sargazi, Shahram Banaie, Mahdi Asani, Hassan Mehrad Majd, Seyed Omid Mohammadi, Alireza Maleki

**Affiliations:** 1 Ophthalmology, Zahedan University of Medical Sciences, Zahedan, IRN; 2 Clinical Research Development Unit, Ghaem Hospital, Mashhad University of Medical Sciences, Mashhad, IRN; 3 Ophthalmology, Burnett School of Medicine, Texas Christian University, Fort Worth, USA

**Keywords:** diabetic eye disease, anti-vegf treatment, bevacizumab, macular edema, dorzolamide

## Abstract

Background: Intravitreal injection of anti-vascular endothelial growth factor (VEGF) agents is accepted as the gold standard treatment for center-involving diabetic macular edema (CI-DME). Adjunctive administration of topical dorzolamide may enhance the therapeutic effects of anti-VEGF agents. In this study, we compared the efficacy of topical dorzolamide plus intravitreal injection of bevacizumab (IVB) versus IVB alone in patients with bilateral DME.

Methods: This prospective, randomized contralateral eye study was carried out in a tertiary referral ophthalmology center, Al-Zahra Eye Hospital, Zahedan, Iran, between April 2021 and April 2022. This study included 50 eyes of 25 patients with bilateral DME. All eyes received three consecutive monthly injections of IVB. For each patient, one eye was randomized to instill dorzolamide eye drops three times a day as an intervention, and the other received artificial tear drops as a placebo. Best-corrected visual acuity (BCVA), central macular thickness (CMT), and intraocular pressure (IOP) were evaluated before starting treatment and then monthly for the first three months.

Results: Among 25 included patients, the average age was 56.64 ± 7.97 years, and 48% were female. BCVA did not improve significantly in any groups (P > 0.05). No significant difference was observed in terms of BCVA between the intervention and control groups (P > 0.05). The present study showed a decrease in CMT in both study groups (P < 0.05). At month 3, the decrease in mean CMT from baseline was significantly higher in eyes receiving topical dorzolamide compared to the control group (-88.92 ± 82.90 vs. -37.64 ± 86.16 µM, respectively; P = 0.037). IOP decreased significantly only in eyes receiving dorzolamide (P < 0.001).

Conclusions: The results of the present study indicate that adjunctive administration of topical dorzolamide has a beneficial effect on CMT reduction from baseline, but it did not have an additive effect on BCVA improvement compared to IVB monotherapy.

## Introduction

Diabetic macular edema (DME) is the major cause of preventable visual loss in the world [1]. Various treatment modalities have been implemented to treat DME. Intravitreal injection of anti-vascular endothelial growth factor (VEGF) agents has been regarded as the gold standard treatment as the first option for center-involving DME (CI-DME) [2]. However, in some cases of DME, the treatment with anti-VEGF agents tends to be unsatisfactory [3]. Thus, the research for other treatment options goes on.

Dorzolamide is a topical carbonic anhydrase inhibitor (CAI) that is primarily used in the treatment of glaucoma [4]. Recent studies pointed out the effectiveness of dorzolamide in the treatment of macular edemas of various etiologies [5-10]. However, the data concerning its efficacy in the treatment of DME are controversial [11-13]. It was reported that adjuvant administration of topical timolol-dorzolamide with intravitreal bevacizumab (IVB) injection might be beneficial in reducing central macular thickness (CMT) [13]. A more recent study reported that adjuvant administration of topical dorzolamide with IVB injection had no additive effects on the treatment of DME [12].

Considering the conflicting results of previous studies on the effects of CAIs in the treatment of DME, as well as the uncertainty around the impact of the adjuvant use of topical dorzolamide on the efficacy of IVB injection, in the present study, we aimed to compare adjuvant administration of topical dorzolamide versus IVB monotherapy in DME. This article was previously posted to the ResearchSquare preprint server on August 10, 2023.

## Materials and methods

This prospective, randomized contralateral eye study was conducted in a tertiary referral ophthalmology center, Al-Zahra Eye Hospital, Zahedan, Iran, between April 2021 and April 2022. A written informed consent was obtained from all participants. The study adhered to the tenets of the Declaration of Helsinki and was approved by the Zahedan University of Medical Sciences Ethics Committee (identifier: IR.ZAUMS.REC.1400.305).

The study was conducted on 50 eyes of 25 treatment-naive diabetic patients with bilateral CI-DME (CMT > 250 μm). The exclusion criteria were as follows: (1) history of any previous laser photocoagulation and/or intraocular surgery/injection, (2) pregnancy or breastfeeding, (3) non-center-involving DME (NCI-DME), (4) history of glaucoma, (5) presence of any other retinal diseases affecting the macula other than DME, (6) history of allergy to the CAIs, and (5) age below 18 years. Meanwhile, patients who failed to attend follow-up meetings or experienced unbearable side effects were excluded.

All eyes were treated with three consecutive monthly intravitreal injections of 1.25 mg/0.05 mL bevacizumab (Avastin, Genentech Inc., San Francisco, CA, USA). All injections were performed in the operating room under sterile conditions. After applying topical tetracaine 0.5% (Anestocaine, Sina Darou, Tehran, Iran), the periocular skin and lid margins were wiped with povidone-iodine 10%. An eyelid speculum was then inserted, and povidone-iodine 5% was applied to the conjunctival sac for three minutes and then washed with sterile saline. Injections were performed using the straight injection technique with a 30-gauge needle through the supratemporal quadrant of the eye approximately 3.5 to 4 mm posterior to the limbus. After injection, a sterile cotton swab was placed over the injection site to avoid reflux and subconjunctival hemorrhage. Then, prophylactic topical antibiotics were prescribed.

For each patient, one of the eyes was randomly allocated to receive topical dorzolamide (Dorzamid 2%, Sina Darou, Tehran, Iran) three times daily. An artificial tear drop (Tearlose, Sina Darou, Tehran, Iran) was prescribed as a placebo for the contralateral eye. The drops' labels were removed to unify their shape, and the drops were coded with random three-digit numbers. Both patients and investigators were blinded to treatment groups.

All eyes were assessed at baseline, one month, two months, and three months after the initiation of IVB treatment. Best-corrected visual acuity (BCVA), central macular thickness (CMT), and intraocular pressure (IOP) were recorded at each appointment. CMT was measured using spectral-domain optical coherence tomography (SD-OCT; Spectralis, Heidelberg Engineering, Heidelberg, Germany). To facilitate statistical analysis, the decimal BCVA was converted to the logarithm of the minimum angle of resolution (logMAR) units. The IOP was measured with Goldmann applanation tonometry.

The statistical analysis was performed using IBM SPSS Statistics for Windows, version 23 (released 2015; IBM Corp., Armonk, New York, United States). Quantitative data were described using mean and standard deviation, and qualitative data were described using frequency and percentage. Based on the normality status of quantitative variables, independent sample t-test and repeated measures ANOVA were used for between-group and within-group analyses, respectively. A level of P < 0.05 was considered statistically significant.

## Results

A total of 50 DME-affected eyes (25 people) were examined. Thirteen patients were male (52%), and 12 patients were female (48%). The mean age of the participants was 56.64 ± 7.97.

Table 1 presents CMT values at baseline and during follow-ups. Both groups showed a significant reduction in CMT during follow-ups (P < 0.05). Although there was no significant difference in CMT between the groups at any time after the initiation of treatment (P > 0.05), the amplitude of the mean CMT reduction from baseline to month 3 was statistically greater in the intervention group compared with the control group (-88.92 ± 82.90 vs. -37.64 ± 86.16 µM, respectively; P = 0.037).

**Table 1:** CMT outcomes in study groups SD: standard deviation, IVB: intravitreal bevacizumab, CMT: central macular thickness, ΔCMT: (CMT third month - CMT baseline)

	Between-group P-value	IVB + placebo	IVB + dorzolamide
		Mean ± SD	Mean ± SD
Baseline	0.808	420.76 ± 148.91	430.52 ± 132.52
1st month	0.673	412.52 ± 128.35	429.04 ± 145.80
2nd month	0.159	405.76 ± 126.42	362.76 ± 81.13
3rd month	0.188	383.12 ± 128.62	341.60 ± 87.13
Within-group P-value		P = 0.047	P < 0.001
ΔCMT	0.037	-37.64 ± 86.16	-88.92 ± 82.90

Table 2 illustrates BCVA values in the present study. BCVA did not change significantly in any treatment groups (P > 0.05). No statistically significant difference in BCVA was identified between groups (P > 0.05), and also there was no significant difference in BCVA improvement between study arms (P = 0.758).

**Table 2:** BCVA outcomes in the study groups SD: standard deviation, IVB: intravitreal bevacizumab, BCVA: best-corrected visual acuity, ΔBCVA: (BCVA third month - BCVA baseline)

	Between-group P-value	IVB + placebo	IVB + dorzolamide
		Mean ± SD	Mean ± SD
Baseline	0.896	0.466 ± 0.283	0.478 ± 0.335
1st month	0.771	0.443 ± 0.271	0.467 ± 0.305
2nd month	0.971	0.440 ± 0.287	0.437 ± 0.305
3rd month	0.969	0.424 ± 0.290	0.427 ± 0.293
Within-group P-value		0.108	0.065
ΔBCVA	0.758	-0.042 ± 0.734	-0.050 ± .112

Table 3 presents the IOP results at baseline and in months 1, 2, and 3. A significant reduction in IOP was observed in the dorzolamide group (P < 0.001). Although not significantly different at baseline (P = 0.112), the IOP was significantly lower in the dorzolamide group at all appointments after initiating the treatment (P < 0.001), and there was a significant difference in IOP changes between the study groups (P < 0.001).

**Table 3:** IOP outcomes in the study groups SD: standard deviation, IOP: intraocular pressure, IVB: intravitreal bevacizumab, ΔIOP: (IOP third month - IOP baseline)

	Between-group P-value	IVB + placebo	IVB + dorzolamide
		Mean ± SD	Mean ± SD
Baseline	0.112	14.32 ± 1.573	15.04 ± 1.567
1st month	P < 0.001	14.52 ± 1.558	12.56 ± 1.660
2nd month	P < 0.001	14.56 ± 1.557	12.32 ± 1.180
3rd month	P < 0.001	14.32 ± 1.773	12.08 ± 1.115
Within-group P-value		0.559	P<0.001
ΔIOP	P < 0.001	0.00 ± 0.764	-2.96 ± 0.935

There were no serious ocular adverse events in either group.

## Discussion

The present study investigated the effects of adjuvant topical dorzolamide in combination with IVB in treating DME. The results of this study demonstrate the significant positive role of dorzolamide administration as an adjunctive treatment in reducing CMT but not in improving BCVA. It should be noted that the administration of dorzolamide caused a significant decrease in IOP compared to placebo.

Several studies have pointed out topical dorzolamide's efficacy in treating cystoid macular edema (CME) [14]. There are several theoretical frameworks regarding the effectiveness of dorzolamide in reducing macular edema. CAIs, including dorzolamide, might directly increase fluid transport across the RPE to improve macular edema by affecting RPE and Müller cells [14]. Dorzolamide also seems to increase the blood flow in the choroid [15]. In addition, CAIs cause acidification of the subretinal space, which may effectively improve CME [16]. Furthermore, there is evidence that the administration of dorzolamide might reduce the production of interleukin-6, an inflammatory cytokine [6].

CAIs might also alter anti-VEGF clearance by their aqueous suppressant activity. Studies investigating the adjuvant administration of dorzolamide in combination with anti-VEGF reported positive therapeutic effects of dorzolamide in macular edema [8-10,13]. It was proposed that dorzolamide could be ineffective in treating DME due to differences in the pathophysiology of DME compared to other types of macular edema. It was found that macular edemas caused by RPE dysfunction and outer retinal diseases are more easily treated with CAIs than those caused by blood vessel leakage and primary retinal vascular diseases [16,17]. Beta-blocker drugs may affect the eyes and macular edema differently compared to CAIs. An animal study showed a decrease in the overproduction of VEGF by systemic administration of propranolol in a mouse model of oxygen-induced retinopathy [18]. Moreover, the beneficial role of topical timolol as a beta-blocker in the treatment of NCI-DME has been demonstrated [19]. In addition, the effectiveness of topical brimonidine as a neuroprotective agent in the management of diabetic maculopathy with ischemic changes has been proposed [20]. In this study, we learned that the addition of topical dorzolamide to IVB injection has a statistically significant impact on reducing CMT but not on improving BCVA. It should also be noted that the administration of dorzolamide was associated with a significant decrease in IOP compared to the placebo.

Badawi et al. suggested that topical dorzolamide without IVB injection can effectively reduce CMT and improve BCVA [11]. Three other studies have investigated the efficacy of topical aqueous suppressants combined with intravitreal anti-VEGF injection in treating DME [12,13,21]. Fazel et al. showed that the adjuvant administration of topical dorzolamide was ineffective in improving the therapeutic effects of IVB injection [12]. Mirshahi et al. reported a decrease in CMT and an improvement in BCVA by adding timolol-dorzolamide eye drop to IVB injection [13]. In 2022, a prospective cohort study indicated that adding topical dorzolamide-timolol as an adjuvant of intravitreal ranibizumab injection (IVR) has no statistically significant benefits [21].

The limitations of this study were the short follow-up period and the small sample size. Theoretically, intervention with dorzolamide could have delayed effects that would make an impact over a more extended period, and thus, the short follow-up time could fail to capture the complete therapeutic effect of the intervention. The small sample size could also limit the generalizability of the findings.

## Conclusions

DME is a complication of diabetic retinopathy. The condition arises from the swelling of the macula due to damaged blood vessels caused by chronic hyperglycemia in diabetes. Key risk factors include poor glycemic control, hypertension, dyslipidemia, and the duration of diabetes. Common symptoms of this condition include blurred and distorted vision. To diagnose this condition, a fundoscopic exam and OCT are often used, while fluorescein angiography may be used in certain cases. Treatment options may include laser photocoagulation and intravitreal injections of anti-VEGF agents or corticosteroids, and in advanced cases, vitrectomy may be necessary. This study aimed to investigate the impact of adjuvant use of topical dorzolamide on the efficacy of IVB injections in DME, addressing the conflicting results from previous studies on the effects of CAIs in treating DME.
